# Supplementary material for: New Diterpenes from Arenga pinnata (Wurmb.) Merr. Fruits
Source: Molecules. 2018 Dec 27;24(1):87. doi: 10.3390/molecules24010087 (PMC6337613; doi:10.3390/molecules24010087)
Supplement: Supplementary file 1 [file molecules-24-00087-s001.pdf]

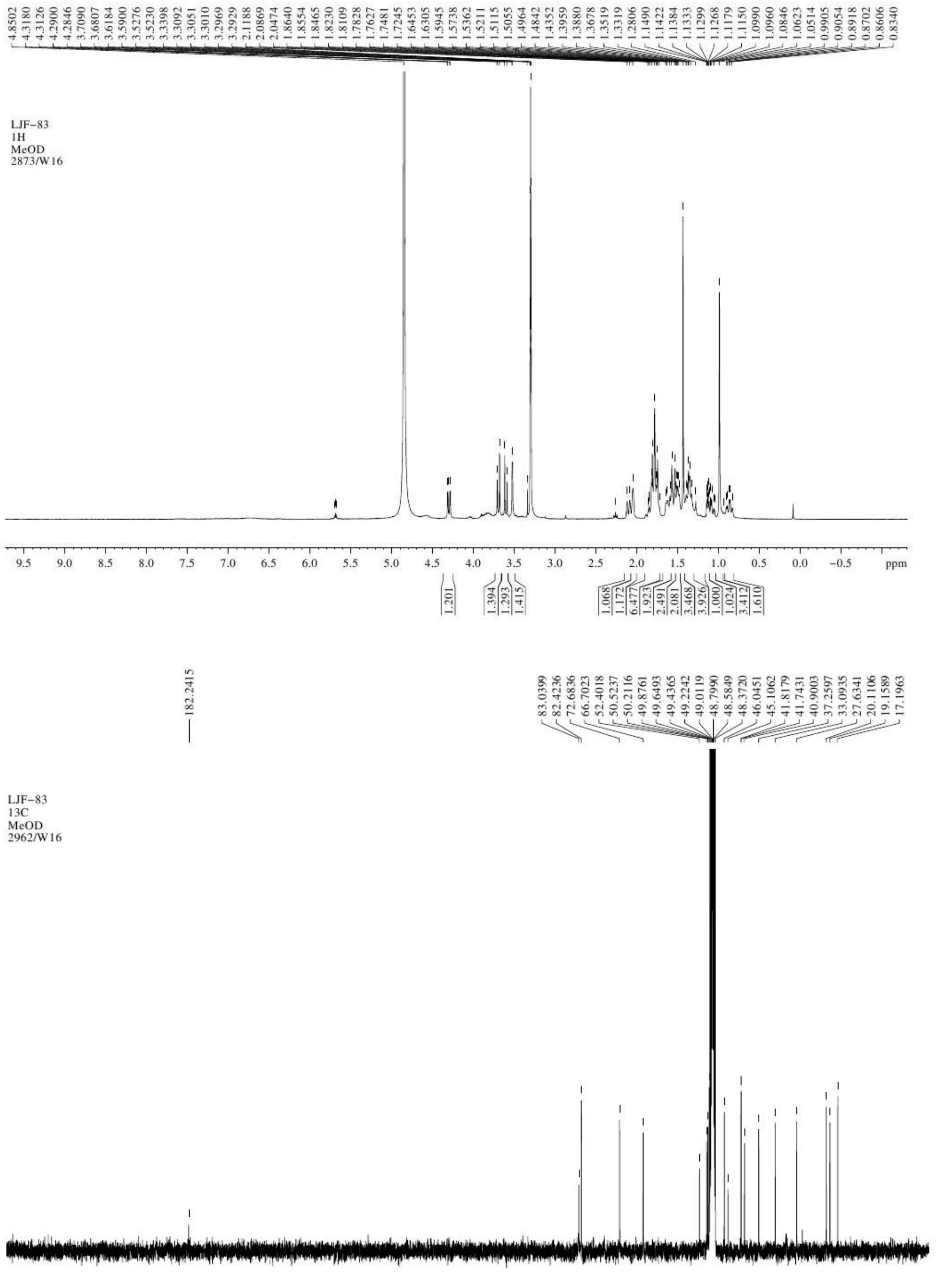

Figures S1 ^{1}H- and ^{13}C-NMR Data of **4** (CD_3OD).

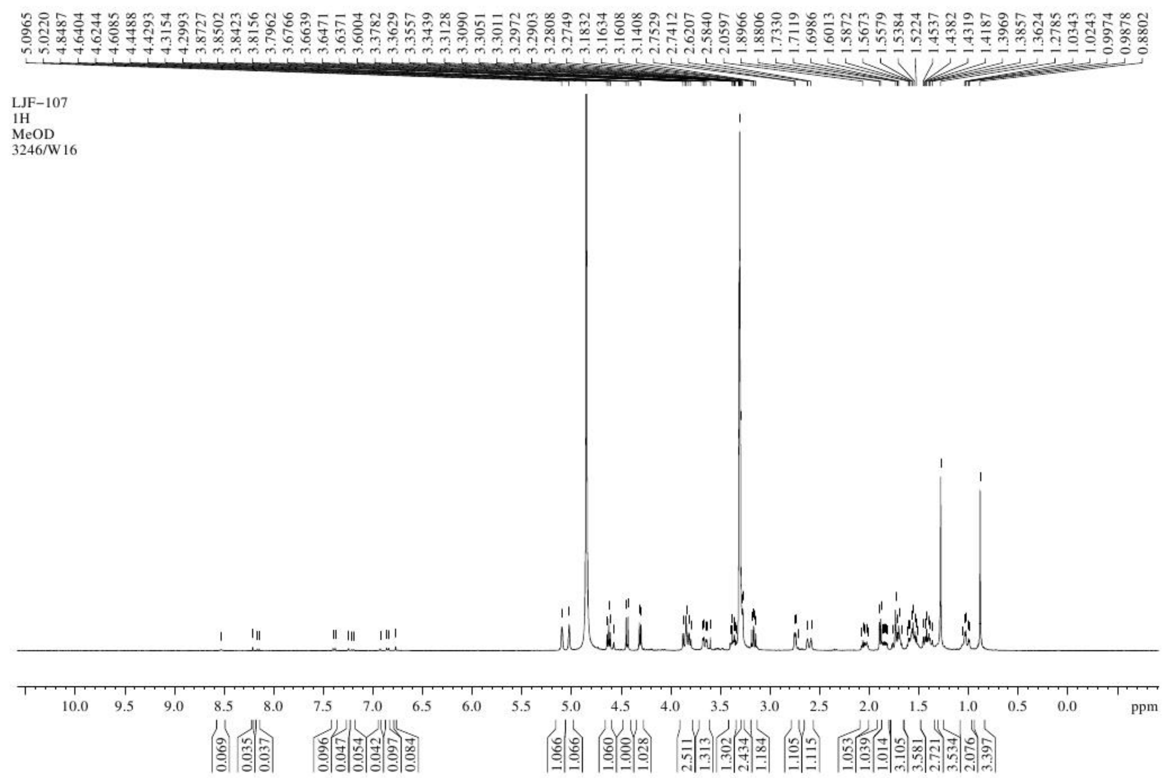

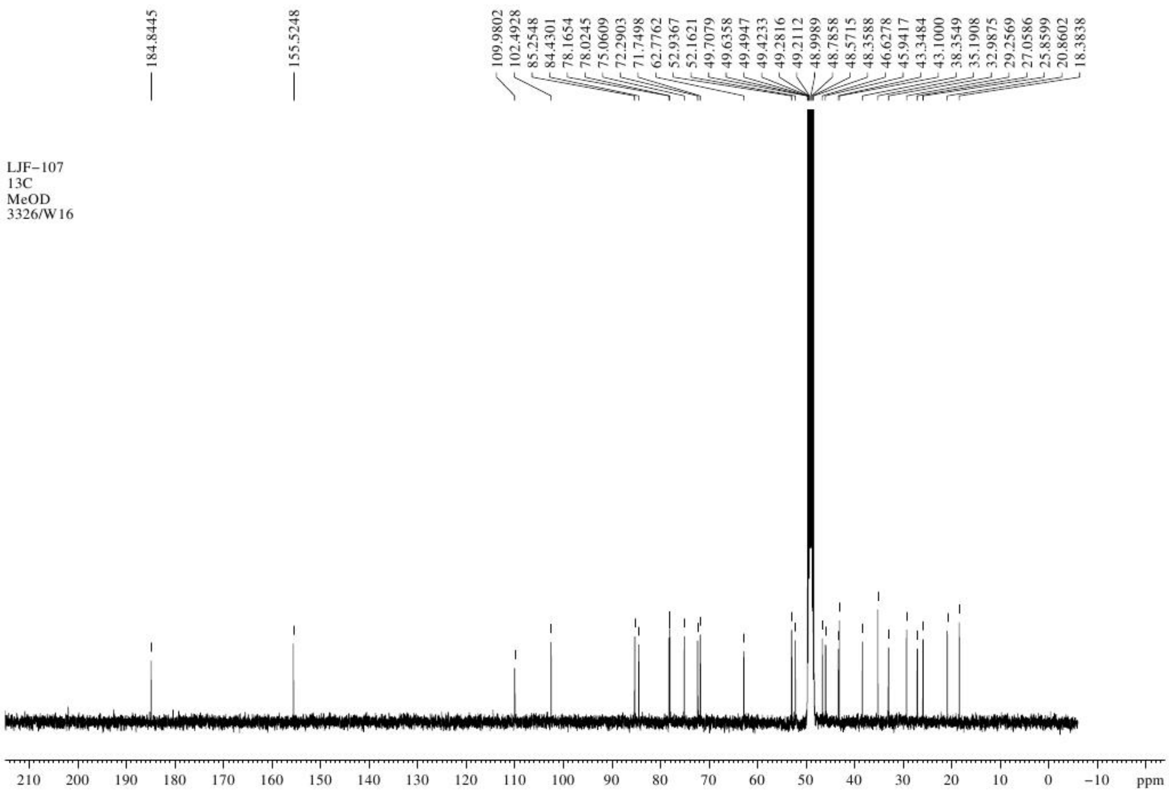

Figures S2 ^{1}H- and ^{13}C-NMR Data of **5** (CD_3OD).

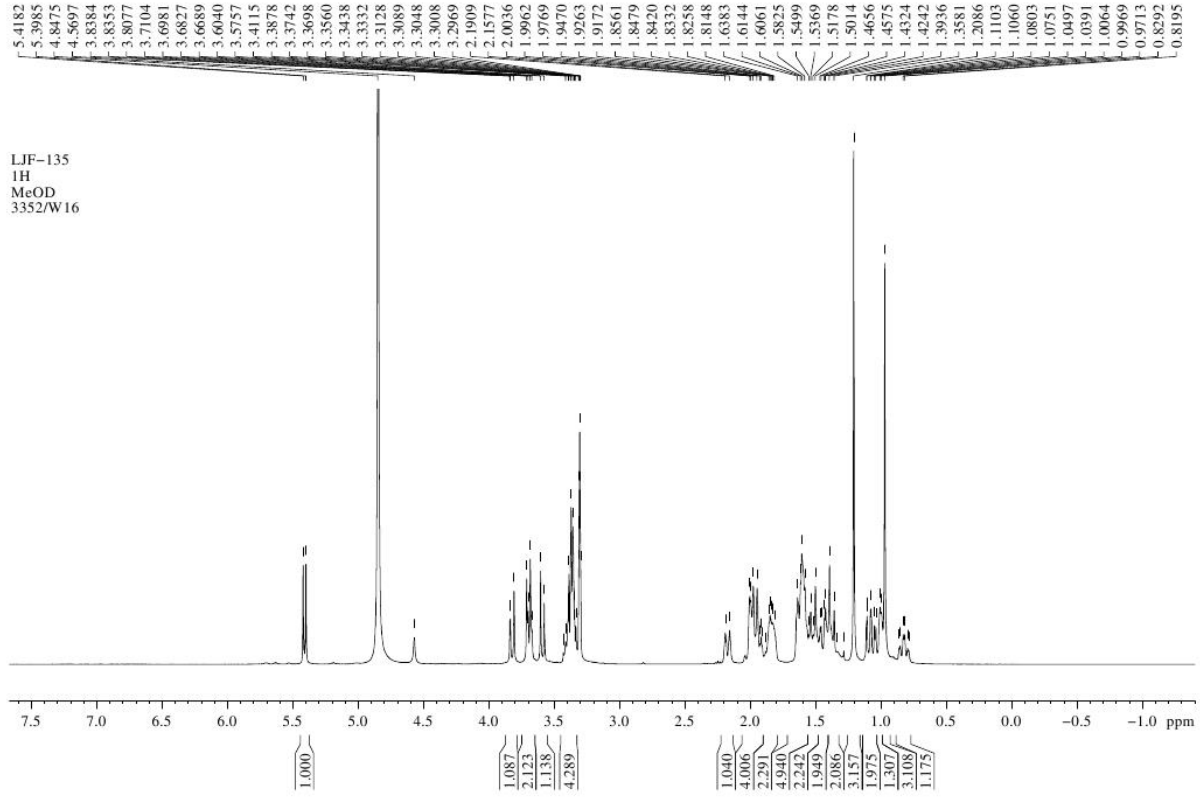

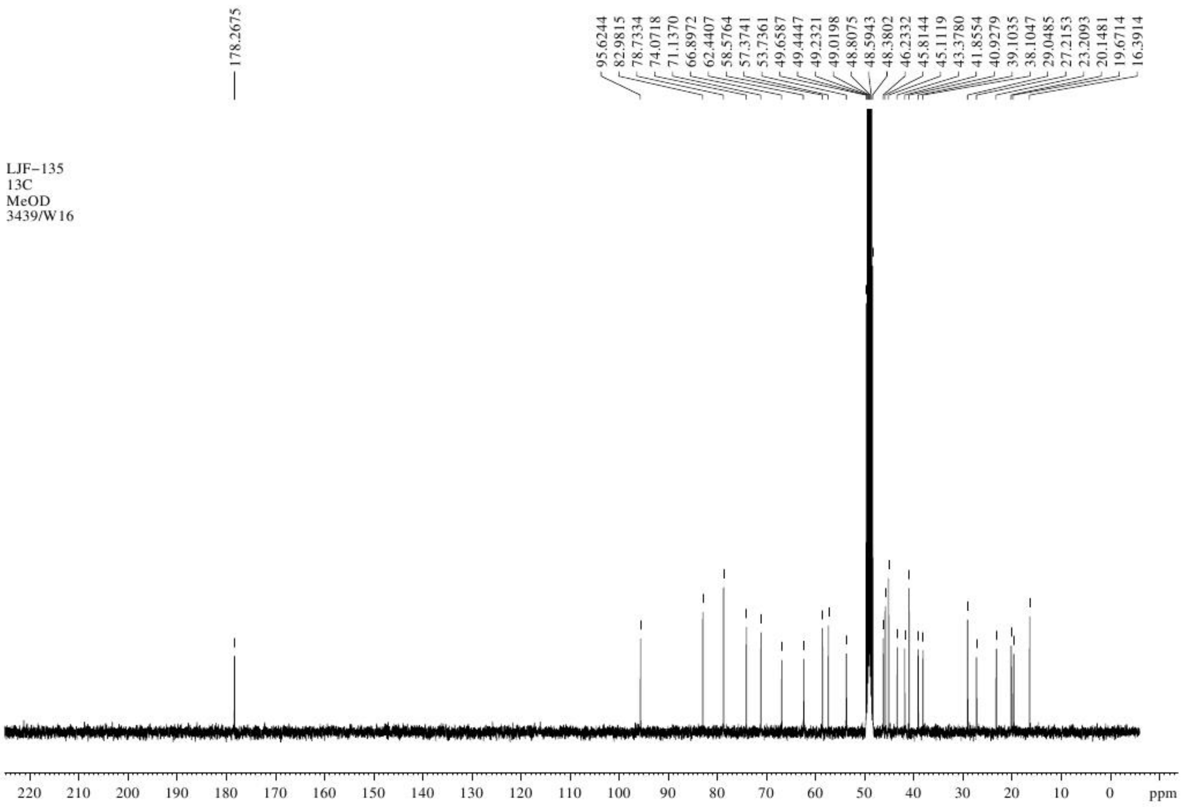

Figures S3 ^{1}H- and ^{13}C-NMR Data of **6** (CD_3OD).

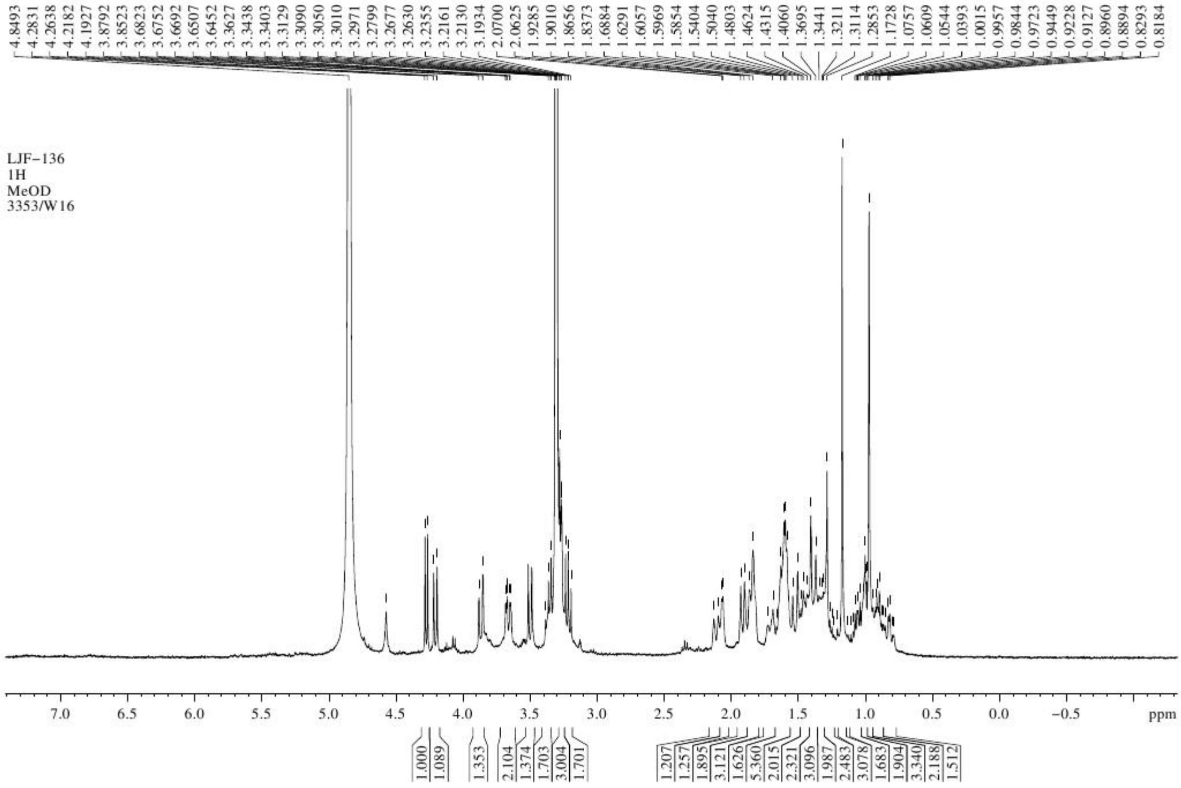

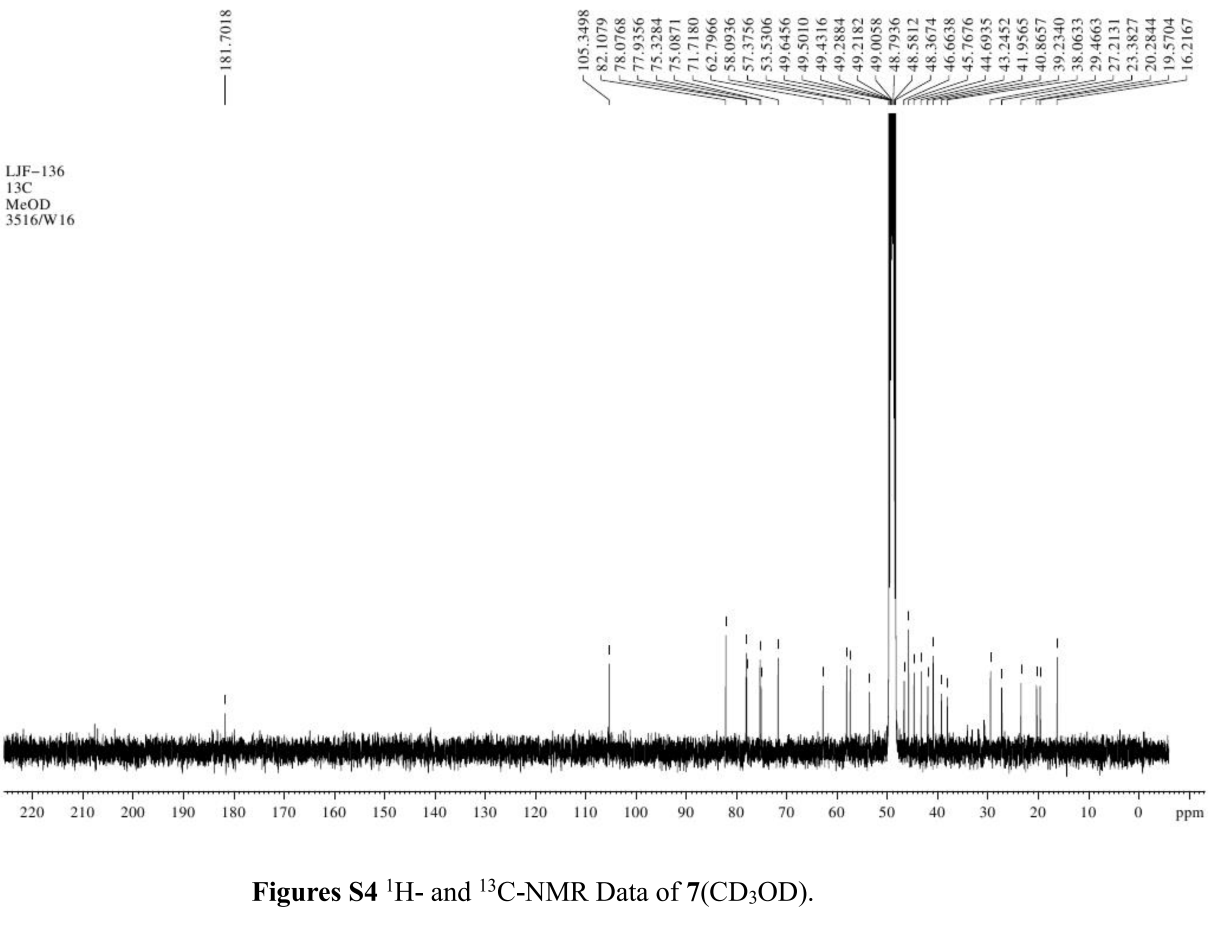

Figures S4 ^{1}H- and ^{13}C-NMR Data of **7**(CD_3OD).

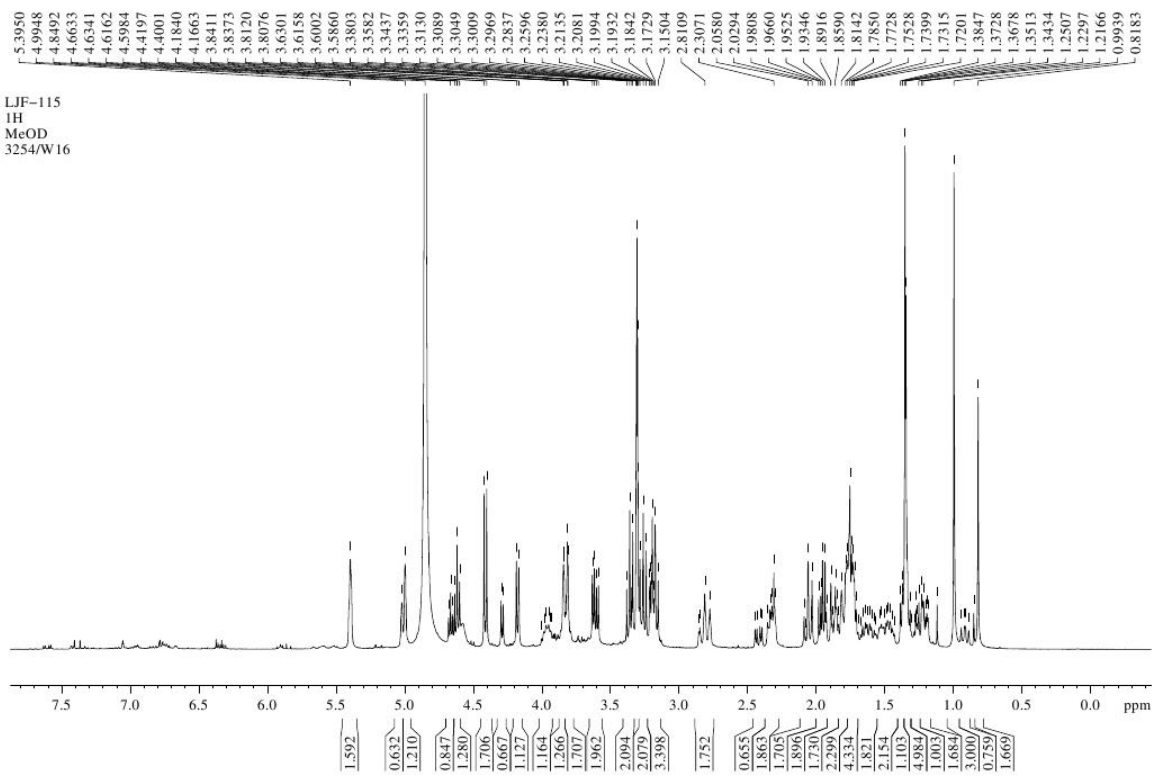

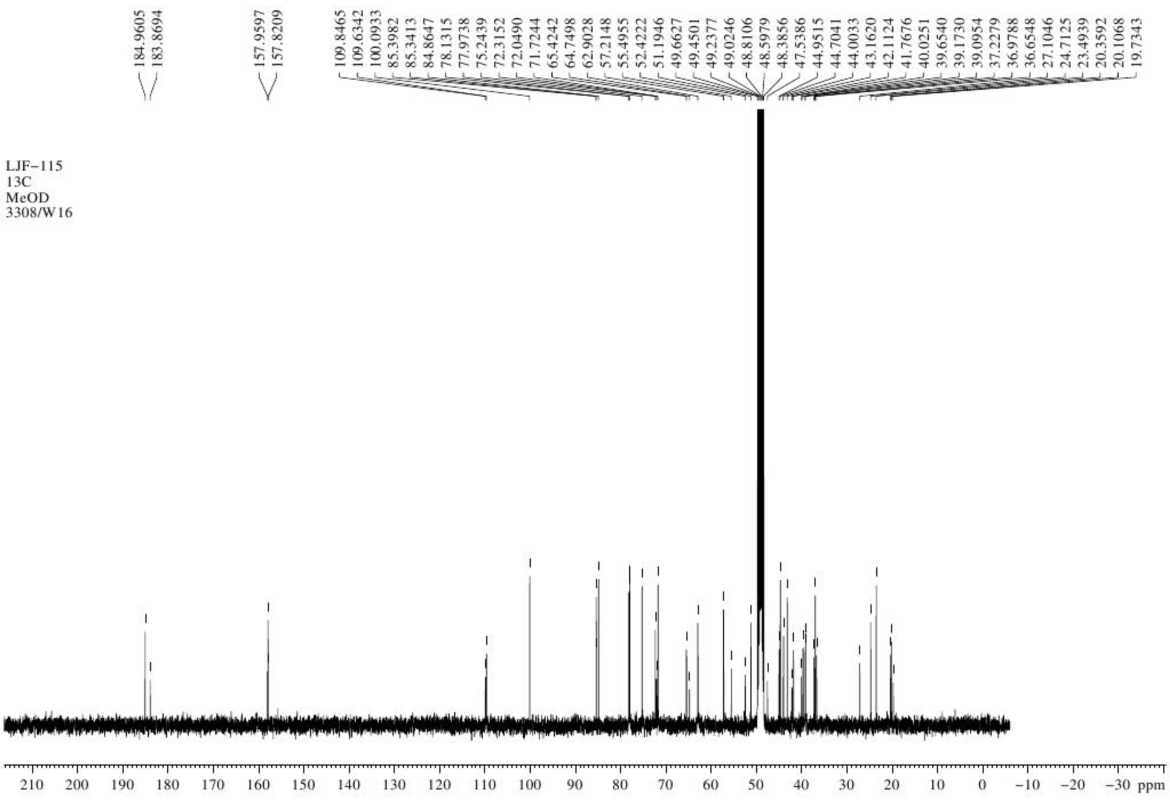

Figures S5 ^{1}H- and ^{13}C-NMR Data of **8** (CD_3OD).

Table S1. ^{13}C-NMR Data of **4–8** (CD_3OD).

NO.	**4**	**5**	**6**	**7**	**8**
	**δ$_C$**	**δ$_C$**	**δ$_C$**	**δ$_C$**	**δ$_C$**
1	41.7	38.4	41.9	42.0	37.0
2	20.1	18.4	20.1	19.6	20.1
3	40.9	29.3	39.1	39.2	39.1
4	45.1	43.1	45.1	44.7	43.2
5	52.4	52.9	58.6	58.1	51.2
6	72.7	84.4	23.2	23.2	84.9
7	83.0	72.3	43.4	43.2	72.3
8	49.9	46.6	45.8	45.8	44.7
9	50.2	52.2	57.4	57.4	57.2
10	41.8	35.2	40.9	40.9	37.2
11	19.2	27.1	19.7	20.3	19.7
12	27.6	84.4	27.2	27.2	27.1
13	82.4	45.9	46.2	46.7	85.4
14	50.5	33.0	38.1	38.1	41.8
15	37.3	43.3	53.7	53.5	39.7
16	46.0	155.5	83.0	82.1	157.8
17	66.7	110.0	66.9	75.1	109.6
18	33.1	184.8	29.0	29.5	24.7
19	182.2	25.9	178.3	181.7	184.9
20	17.2	20.9	16.4	16.2	23.5
1'		102.5	95.6	105.3	100.1
2'		75.1	74.1	75.3	75.2
3'		78.2	78.7	78.1	78.1
4'		71.7	71.1	71.7	71.7
5'		78.0	78.7	77.9	78.0
6'		62.8	62.4	62.8	62.9
